# Potential of acute phase proteins as predictor of postpartum uterine infections during transition period and its regulatory mechanism in dairy cattle

**DOI:** 10.14202/vetworld.2016.91-100

**Published:** 2016-01-29

**Authors:** A. Manimaran, A. Kumaresan, S. Jeyakumar, T. K. Mohanty, V. Sejian, Narender Kumar, L. Sreela, M. Arul Prakash, P. Mooventhan, A. Anantharaj, D. N. Das

**Affiliations:** 1 Southern Regional Station, ICAR - National Dairy Research Institute, Adugodi, Bengaluru - 560 030, Karnataka, India; 2 Theriogenology Laboratory, ICAR - National Dairy Research Institute, Karnal-132 001, Haryana, Uttar Pradesh, India; 3 ICAR - National Dairy Research Institute, Karnal - 132 001, Haryana, India

**Keywords:** cute phase proteins, bovine uterine infections, pro-inflammatory cytokines, negative energy balance, transition period

## Abstract

Among the various systemic reactions against infection or injury, the acute phase response is the cascade of reaction and mostly coordinated by cytokines-mediated acute phase proteins (APPs) production. Since APPs are sensitive innate immune molecules, they are useful for early detection of inflammation in bovines and believed to be better discriminators than routine hematological parameters. Therefore, the possibility of using APPs as a diagnostic and prognostic marker of inflammation in major bovine health disorders including postpartum uterine infection has been explored by many workers. In this review, we discussed specifically importance of postpartum uterine infection, the role of energy balance in uterine infections and potential of APPs as a predictor of postpartum uterine infections during the transition period and its regulatory mechanism in dairy cattle.

## Introduction

The incidence of uterine infections in cows has been reported between 10% and 50% with 10-20% of metritis, 15% of clinical endometritis, and 15% of subclinical endometritis [1-3]. Sheldon and Dobson [2] reported that up to 40% of the animals have a uterine infection during initial 2 weeks postpartum, in which 10-15% show persisted infection for at least another 3 weeks while 30-35% of cows have subclinical endometritis between 4 and 9 weeks postpartum [4]. The high prevalence of postpartum uterine infections in buffalo cows (38.54%) and in crossbred cows (up to 29.7%) was also reported in India [5,6]. Uterine infections cause inflammation, histological alteration, delayed uterine involution, reduced pituitary LH secretion, and disruption of ovarian follicular growth and its function in dairy cattle [7]. Dubuc *et al*. [8] found 3.7 kg loss of milk production per day in multiparous cows due to metritis. When considering reproductive parameters alone, reproductive inefficiency beyond 100 days postpartum results in an estimated loss of $2.5 to $3 per cow per day, while overall loss due to reproductive inefficiency has been estimated to be $5.4 per cow per day [9]. With approximately 100 dairy cows with 10% incidence of uterine infections, the cost of uterine infections/cow/month would be about $750-$1620.

Although more than 95% of the animals get exposed to bacterial contaminations during calving, only a few of them develop uterine disease, and it could be the associated outcome of pathogens, animal, and the environment. However, in a particular environment with equal chances of exposure to pathogens, the more susceptibility of some animals in the herd suggested the greater importance of animal factor over the environment or pathogen-related factors. In fact, an impairment of immune functions during the peri-partum period has an important role in determining whether an animal develops postpartum uterine disease or not. However, the exact mechanism that initiates and sustains the uterine inflammation is not clear at present. Among the various factors, the energy status of the peri-partum animals is one of the most important determinants for the development of uterine disease. Endocrine and metabolic changes around parturition are believed to depress the uterine defense mechanism, which favors the development of uterine disease in dairy cattle [10,11]. The first response of the innate immune system against uterine infection is the invasion of neutrophils into the uterus. However, the invasion of neutrophils is determined by pro-inflammatory cytokines and other factors. Recently, Galvao *et al*. [12] observed that lower expression of pro-inflammatory cytokines in the endometrium immediately after calving impaired the chemotaxis and activation of neutrophils which leads to the development of endometritis in cows. Further, the functional capacity of neutrophils is determined by many factors, and negative energy balance (NEB) is believed to be an important determinant [13].

Prevention and early treatment of postpartum uterine infections are more economical than treatment at a later stage when diseases get established. Thus, early diagnosis or predictions of uterine infections are important for effective postpartum management. Since the postpartum complications are of multi-factorial nature; it has been difficult to find out the biomarker for early prediction of uterine infections. Recently, many workers [7,14-16] have explored the possibility of bovine major acute phase proteins (APPs) such as haptoglobins (Hp) and serum amyloid A (SAA) as biomarkers to predict the postpartum uterine infections and found different results. Huzzey *et al*. [17] suggested that acute phase response (APR) precedes clinical metritis, and thus Hp screening may assist for early detection of metritis. They [18] also found that Hp concentration tended to be greater during pre-partum cows that developed more than one disorder or that died by 30 days in milk. Dubuc *et al*. [19] reported that Hp concentrations higher than 0.8 g/l during 1^st^ week after parturition are a risk factor for endometritis and purulent vaginal discharge. Although several review regarding the role of APPs in dairy animals are available [20,21], we are specifically discussing here the potential of APPs as the predictor of postpartum uterine infections during the transition period and its regulatory mechanism in dairy cattle.

## Uterine Immunology during Transition Period

Although, our understanding of the role of the uterine immune system is still limited, it is believed that uterine defense mechanisms play a major role during the postpartum period. Cellular defense against bacterial contaminants is mainly provided by uterine leukocytes, in which neutrophils are the major and primary defense molecule involved in bacterial clearance after uterine infection [22]. Neutrophils are short-lived (1-3 days) cells, and they have the ability to migrate and engulf the foreign invaders. They execute their functions through oxygen-dependent free radical-mediated killing (respiratory burst) or through enzymatic killing of the pathogens. It is believed that adequate recruitment of functionally active neutrophils in the uterus is foremost important for clearing the bacteria [22]. In general, pathogen recognition receptors on the endometrial cells and macrophages mainly toll-like receptors, recognize the pathogen through pathogen-associated molecular patterns and secrete or release the mediators such as pro-inflammatory cytokines (interleukin [IL]-1β, IL-6, tumor necrosis factor-α [TNF-α], etc.) and chemokines (IL-8) [23], then IL-6, TNF-α, and IL-8 stimulate the production of antimicrobial peptides by endometrial cells or accelerate the polymorphonuclear (PMN) cells infiltration into endometrium for elimination of pathogens [24]. The function of TNF-α is to stimulate the IL-8 expression and cell adhesion molecules on vascular endothelium while IL-8 is a potent chemoattractant. However, the kinetics of neutrophils with reference to the magnitude of movement and time of appearance in the uterus depends on chemoattractant produced by inflammation or bacterial stimulation, energy state, hormonal influence, and other factors. For instance, Zerbe *et al*. [25] reported that infusion of human recombinant IL-8 into bovine uterus cause attraction of PMN, while anti-IL-8 antibody prevented the PMN-dependent PMN infiltration and subsequent tissue damage. Although, this observation confirmed the IL-8 role in normal animals, its role under the influence of uterine infections remains to be confirmed. They also suggested the influence of bacteria and its components on neutrophils entry into uterus [26]. Although, the cytokines are believed to play an important role in neutrophil migration and clearance of pathogens, higher or excessive expressions of pro-inflammatory cytokines are often associated with greater inflammation during the 1^st^ or 2^nd^ week postpartum [27,28], while their lower expression in the endometrium immediately after calving impaired the chemotaxis and activation of neutrophils which leads to development of endometritis in cows [12]. Collectively, it suggests that adequate stimulation of pro-inflammatory cytokines is critical for the healthy uterus and any changes in these cytokines synthesis will adversely affect the uterine immunity.

Several authors suggested that changes in cytokines levels during the peri-partum period could be useful for predicting postpartum complications. Ishikawa *et al*. [29] investigated the correlation between the IL-6 concentration and the occurrence of postpartum diseases and found that cows suffered with endometritis had a higher level of pre-partum IL-6 than control. They suggested that alteration in the IL-6 concentration during pregnancy was one the useful tool for predicting postpartum diseases in dairy cattle. Kim *et al*. [11] reported that dairy cows suffered with endometritis during the 3^rd^ and 4^th^ week postpartum period had a higher concentration of TNF-α than normal cows. Islama *et al*. [30] observed the significantly higher concentration of IL-10 in clinical metritis than normal cows at 15 days before calving, at calving and 15 days postpartum. However, the significant difference was found only at 30 d postpartum for clinical endometritis cows. Kasimanickam *et al*. [31] found that cows with metritis or clinical endometritis had higher serum concentrations of IL-1β, TNF-α, and IL-6 compared to normal cows and suggested that loss of body conditions mediated increases in cytokines and thereby prolonged the uterine inflammation in dairy cows.

## Energy Balance and Postpartum Uterine Infections

Among the various factors, the energy status of the postpartum animals is one of the most important determinants for the development of uterine disease. Hammon *et al*. [13] reported that cows in greater NEB have more pronounced impairment of immune functions and susceptibility to develop metritis or endometritis. Among the various mediators, inflammatory cytokines are believed to be as central integrators of metabolic changes and immune function [32], particularly during the transition period. For instances, slower increase of negative APPs such as albumin and higher concentration of NEFA, BHBA in transition cows after administration of interferon alpha [33], increased risk of ketosis in cows administered TNF-α during late pregnancy [34], and possible role of IL-6 in ketosis [35] are suggestive for the role of cytokines in metabolic disorders. Collectively, it suggests that cytokines have an important role during the transition period. NEB-mediated alterations of gene expression and metabolites (NEFA or BHBA) also have an important role in uterine immunity. Beam and Butler [36] observed that cows with severe NEB had increased uterine pro-inflammatory cytokine gene expression at 2 weeks postpartum compared to moderate NEB cows. Further, it was supported by the findings of Wathes *et al*. [37] that NEB caused more expression of uterine inflammation-associated genes (IL-1 and IL-8 receptors). The higher concentrations of NEFA and BHBA during pre-partum have been associated with postpartum metritis and endometritis in cows, and it could be mediated through impairment of neutrophils function [13,38]. Decreased concentrations of glycogen in neutrophils and blood calcium were also suggested for reduction of neutrophil function and thus favor postpartum uterine infection [38]. Recently, Giuliodori *et al*. [39] found that higher pre-partum NEFA and postpartum BHBA levels increased the risk for endometritis, whereas high pre-partum BUN reduced it.

**Figure-1:**
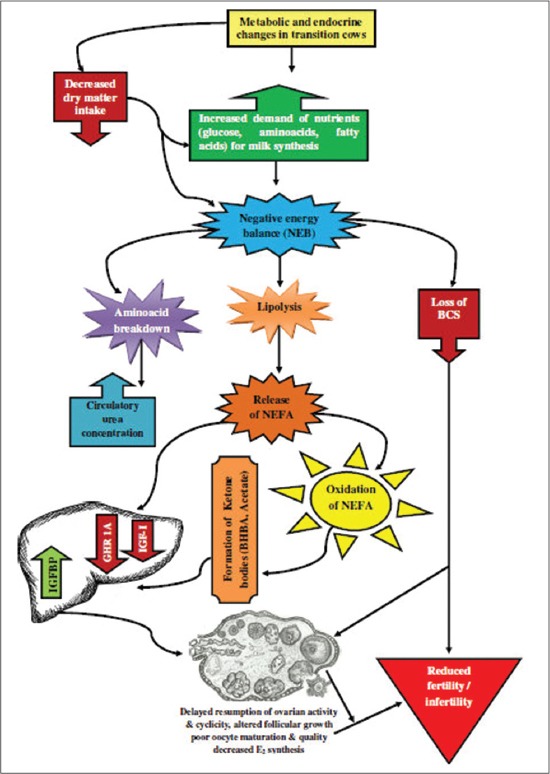
Effects of negative energy balance on reproductive function. Up arrow indicate increase while down arrows indicate a decrease.

Although the major consequence of uterine infection is conception failure and subsequent culling [40], the possible mechanism for such outcome remains unclear. Therefore, proper investigation of the mechanism would be very useful for the development of diagnostic or prognostic markers for postpartum health assessment. Rossi *et al*. [41] reported that NEB can affect cow reproductive performances through metabolic hormonal modifications. They suggested that profound changes in the liver lead to a reduction in the concentration of growth hormone receptor (GHR), insulin-like growth factor (IGF)-I level, IGF binding proteins (IGFBPs), and the acid-labile subunit while IGFBP-2 was increased. Collectively, it causes the impairment of reproductive functions (Figure-1).

### Biomarkers in Uterine Infections

Identification of biomarkers against various diseases becomes a major thrust area of research in this “omic” era due to the discovery of new molecules and technologies. Identification of biomarkers would be useful to assess the pathophysiological status of the animal and thus early diagnosis, treatment, and prevention of economically important diseases including postpartum uterine infections. Though some traditional markers (such as NEFA, BHBA, and feeding behavior) are available to predict the postpartum uterine infections, they were not successful due to its variability between the conditions. Silva *et al*. [42] explored the possible role of COX-2 and PGE_2_ as biomarkers (at transcription and protein level) and found that they were not useful markers. Recently, Dubuc *et al*. [19] suggested that higher pre-partum NEFA concentration, dystokia, and RFM as important risk factors for metritis while, ketosis as a risk factor for subclinical endometritis and dystokia, twinning, and metritis for clinical endometritis. Interestingly, they found that increased Hp concentration during the 1^st^ week of postpartum as a common risk factor for all these conditions [19]. It suggested that despite variation in clinical manifestations of postpartum uterine infections, Hp could serve as a better indicator for the postpartum performance of dairy animals. Cairoli *et al*. [43] found that the concentrations of Hp and α_1_-acid glycoprotein (AGP) were fluctuated at the time of calving and in cows affected with postpartum endometritis.

### Role of APPs in Bovine Reproduction

**Figure-2:**
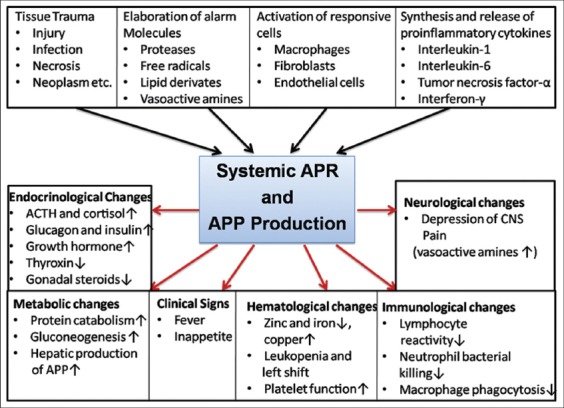
Acute phase responses. ­increase, ¯decrease.

Following infection, injury or even with changes in normal physiological homeostasis, a number of systemic responses take place, and APR is one of them. APR is a cascade of systemic reactions (Figure-2) against inflammation, mostly coordinated by cytokines, which are produced from macrophages or any other inflammatory cells. Production of pro-inflammatory cytokines (mainly IL-1β, TNF-α, and IL-6) at the site of injury, subsequently stimulates the production of APPs from a local site or at the liver. APPs are classified into three categories based on the: (1) magnitude of elevation in blood (positive if they increase or negative if they decrease), (2) time of APPs released into blood in response to APR (first phase proteins are elevated immediately and second phase proteins are increased after 1-3 days), and (3) cytokines which are responsible for its stimulation (IL-1- and IL-6-dependent response). Collectively, it suggests that the kinetics of APPs would differ based on APR, severity of infection and time course of infections. About 40 APPs have been identified in humans. Out of which, nine APPs are well-studied in cattle. Among these nine proteins, Hp and SAA are considered as major, while AGP is moderate and fibrinogen (Fb) is minor [20,44]. Further, the major APPs (Hp and SAA) can also act as moderate APPs in bovines. Several researchers observed that Hp concentrations are often undetectable in healthy cattle [45] but increase about 50±100 times during an APR, making it the most prominent APP in cattle [46]. On the other hand, SAA is a low constitutive APP in cattle increasing around 2±5 times or 10-fold during an APR [46,47]. Nevertheless, SAA seems to react faster than Hp against APR [48], and thus SAA is considered as good marker for acute clinical conditions [49] while Hp as a better marker for chronic conditions [50].

The role of APPs following infection has been extensively evaluated in human practice to monitor pathogenesis of disease and efficacy of treatment. In veterinary practice, the application of APPs for the determination of health status in domestic and pet animals was reviewed by various authors [20,21,45,51]. In large ruminants, the role of APPs in mastitis was extensively studied [52-55]. Krakowski and Zdzisińska [56] reported that major bovine APPs plays an important role in the reproductive processes through intensification of the phagocytosis against the pathogens introduced into the uterus and by the reconstruction of the endometrium. Indeed, the role of APPs in prediction, diagnosis, and treatment evaluation of postpartum uterine infections was also reported by several researchers [18,57-59]. Mordark [60], found a higher concentration of Hp in cows with retained placenta, which is believed to be an immune-mediated disorder. Williams *et al*. [61] observed that cows with high uterine pathogen growth density had higher peripheral concentrations of AGP, SAA, and Hp compared to low uterine pathogen growth density cows during 7 and 14 days postpartum period. A positive correlation between APPs level and severity of disease and the extent of the tissue damage was also reported by Baumann and Gauldie [62]. Further, they suggested the differential kinetics of the APR between APPs, where AGP had more persistent effect than Hp or SAA. In contrast, Pyorala [51] revealed that the AGP levels did not correlate with the severity of disease compared to Hp, and thus the capacity of AGP in differentiating uterine infections was poor.

Measurements of serum Hp and SAA have been widely used to diagnose the uterine infection in postpartum cows. Hirvonen *et al*. [58] studied the diagnostic and prognostic efficacy of Hp and AGP in acute metritis cows during postpartum period and found increased plasma Hp concentration in metritic cows and predicted the poor condition and low fertile cows. Regassa and Noakes [63] reported that presence of intrauterine infections did not affect the uterine involution in mule ewes. However, the Hp level was significantly higher in the uterine infected ewes than the healthy ewes. Sheldon *et al*. [64] found that uterine involution was associated with a decrease in the concentrations of APPs (AGP, Hp, and CP). However, the bacterial contamination increased the APPs irrespective of uterine involution status. It suggested that APPs could be useful to differentiate between postpartum infections with normal physiological events. Horadagoda *et al*. [50] reported that assays of APP, particularly Hp and SAA, might differentiate the chronic from the acute inflammation in cattle in a better way than the hematological tests. In contrast, Humblet *et al*. [14] suggested that Hp and SAA were good markers for identification of healthy animals, but their ability to identify the diseased animals was low as most of the postpartum diseases were chronic in nature. Further, they reported that physiological status of a cow, particularly at parturition, can have an influence on serum Hp concentration. Heidarpour *et al*. [65], found that cows suffered with clinical and subclinical endometritis had significantly higher concentration of Hp. Schneider *et al*. [66] found that cows diagnosed with uterine infection had a higher concentration of Hp during 7 days postpartum. Besides, Kováč *et al*. [67] studied the relationship between APPs (Hp and SAA) and energy metabolites (NEFA and BHBA) in dairy cows and found significant correlations between Hp with NEFA as well as BHBA and SAA with only NEFA. Various workers reported a positive correlation between hormonal status (estrogens, progesterone, and cortisol) and Hp level during the last trimester of pregnancy and after calving [68,69].

Smith *et al*. [57] evaluated the therapeutic efficacy of different antimicrobial regimens through serum Hp concentrations in cows suffered with toxic puerperal metritis and found that the 5-day treatments reduced the serum Hp concentration (19 mg/dl vs. 7.35 mg/dl). Heidarpour *et al*. [65] reported that Hp level was reduced after treatment in cows suffered with clinical endometritis and cows had a lower concentration of Hp (before treatment), shown a better response to further treatment. In contrast, Jeremejeva *et al*. [60] evaluated the effect of two treatment strategies (ceftiofur + NSIAD and ceftiofur + PGF2α) in acute puerperal metritis and clinical metritis cows through APPs (SAA and Hp) and found no significant difference. Mordak [70] found that the cows with highest Hp concentration were expelled placenta after 4 days (2.22 g/l) and cows with the lowest concentration of Hp had been easily removed the placenta (0.9 g/l).

Besides, the diagnostic and prognostic applications of APPs few studies were attempted to see the dynamics of APPs with postpartum performance to consider the potential role of APPs as a predictor for uterine infections and subsequent conception. Chan *et al*. [71] reported that cows suffered by postpartum reproductive disorders had a significantly greater concentration of Hp than the healthy animals with no significant influence of season or pregnancy. It suggests that Hp is not influenced by environment or physiological status and thus a useful indicator for cows with postpartum reproductive disorders. Huzzey *et al*. [17] reported that mild and severe metritis cows had higher Hp concentrations than healthy cows in early postpartum period (between 0 and 12) and cows with ≥1 g/L Hp concentrations on day 3 postpartum were 6.7 times more susceptible for severe or mild metritis with 50% sensitivity and 87% specificity. They suggested that APR precedes clinical metritis, and thus Hp screening may assist for early detection of metritis and opportunities for early treatment or prevention. Chan *et al*. [16] found that serum Hp concentrations in cows suffered with acute puerperal metritis were significantly higher than clinically healthy animals from 1 week pre-partum to 6-month postpartum period. In addition, among the successfully pregnant animals, the number of days open was significantly higher in cows had a higher concentration of Hp than cows with a lower concentration of Hp; suggest that Hp may also be a useful predictor of postpartum reproductive performance. Sabedra *et al*. [72] reported that elevated serum Hp concentrations were associated with disease status (healthy vs. infected), severity (healthy, moderate, severe, or died), number, and type of disease (one or more diseases such as metritis, ketosis and mastitis), birth complications, and clinical onset of disease in early lactation. Further, they suggested that serum Hp concentrations may assist in early detection and treatment of diseases in early lactation. Burke *et al*. [73] reported that among successfully pregnant animals, the number of days open was significantly higher in cows with a high concentration of Hp suggesting its prediction ability of postpartum performance even in the absence of uterine infections.

Recently Huzzy *et al*. [74] evaluated the association between peri-partum (3 weeks before calving to 10 days after calving) markers of stress (cortisol), inflammation (Hp), and energy balance (NEFA and BHBA) and milk yield and reproductive performance in HF cows and found negative association of Hp and other markers with milk yield and reproductive performance. It suggested that understanding of Hp dynamics during peri-partum would be useful for assessing the opportunities for improved milk yield and reproduction. Krause *et al*. [75] evaluated the association between resumption of postpartum ovarian activity, uterine health (PMN cells and P_4_ levels), severity of the NEB (through NEFA, glucose and insulin levels), and the synthesis of inflammatory mediators (albumin and Hp levels and paraoxonase activity) during the transition period and found that cows resumed ovarian activity early in the postpartum period had higher albumin concentrations during peri-partum period. However, they found no association between APPs levels with subclinical endometritis incidence and markers of energy indicators or milk yield with postpartum cyclicity. It suggested that influences of energy on APPs dynamics need to be further evaluated. Nightingale *et al*. [76] investigated the relationship between the intensity of the APR (through classification of cows with low, medium and high concentrations of Hp) and the metabolic status and leukocyte responses of early postpartum cows and found that postpartum reproductive performance was impaired in cows with a greater APR (days open in these groups were 123, 139, and 183 days, respectively). They suggested that a stronger APR during the early postpartum period is characterized by an activated innate immune system, and a suppressed mitogen-induced interferon-γ secretion resulted in impaired reproductive efficiency.

## APPs and Cytokines Expression in Uterine Tissues

APPs are primarily synthesized by the liver against APR. However, there is increasing evidence for the extra-hepatic expression of APPs for the local needs. For instance, the APP synthesis in the mammary gland is a well-known phenomenon during mastitis or physiological changes such as involution. Chapwanya *et al*. [28] reported that expression of SAA3 mRNA was increased in early postpartum (2 weeks) compared to late postpartum (9 weeks) cows and SAA reflected the severity of inflammation. They also revealed that the SAA expression was higher than Hp mRNA expression. Gabler *et al*. [77] revealed time-related expression of inflammatory cytokines and APPs, with significant peak expression on the day 17 postpartum period as a possible mucosal immune response in the uterus. In addition, they found that IL-1β, IL-8, and Hp mRNA expression were correlated significantly with the proportion of PMN. Collectively, it suggests that the dynamics of these APPs differs between normal and inflamed uterus and it needs further investigation. Further, the moderate level of expression of AGP in the healthy uterus has been reported by some researchers [78,79], but there is no information on AGP expression in the infected uterus.

Fischer *et al*. [24] found that the expression of IL-1β, IL-8, and TNF- α mRNA was significantly higher in cows with subclinical or clinical endometritis compared with healthy cows while there was no indication or correlation with uterine health for IL-6 and Hp transcripts. They suggested that IL-1β, IL-8, and TNF-α might represent potential marker genes for the detection of cows with subclinical endometritis and for monitoring new therapeutic approaches. Ghasemi *et al*. [80] found 20, 30, and more than 50-fold higher mRNA expression of TNF-α, IL-6, and IL-8 level, respectively, in subclinical endometritis than healthy cows. They suggested that IL-8 gene expression might be useful to predict endometrial inflammation. Galvão *et al*. [81] found that *Escherichia coli*-stimulated monocytes from cows with metritis had lower expression of TNF-α, IL-1β, and IL-6 than healthy counterpart while there were no significant differences in IL-8 or IL-10 expression in these cows. Collectively, it suggested that pro-inflammatory cytokines expressions are differentially altered during postpartum uterine infections.

Loyi *et al*. [82] found several fold higher expression of cytokines (IL-1β, IL-6, IL-8, and TNF-α) in endometritic samples while significant up-regulation of CD14, IL-6, IL-8, and TNF-α mRNA in subclinical endometritic buffaloes samples collected from the abattoir. Chapwanya *et al*. [83] found that expression of IL-1β, TNF-α, and SAA3 genes were increased by 121-, 357-, and 721-fold, respectively, during 6 h post *E. coli* stimulation. However, IL-1β, IL-6, IL-8, and TNF-α gene expression was decreased, whereas SAA3 expression was further increased to 3452-fold after 24 h of *E. coli* stimulation compared to 6 h. They suggested that better understanding of localized endometrial expression of SAA3 was needed to use SAA as a sensitive diagnostic marker for *E. coli* infection in cattle.

## Transcriptional Regulation of APP Production

Although, APR and subsequent changes in serum concentrations of APPs are known for almost a century, the biological importance of the different APPs and molecular mechanisms controlling their expression is just beginning to emerge. The expressions of APPs in hepatocytes are mostly controlled at the transcriptional level by different transcriptional factors such as signal transducer and activator of transcription (STAT) family [84] and NF-kB [85]. However, the transcription factors involved in the regulation of each APPs and in species are differed. For instance, the transcriptional regulation of SAA gene is mainly controlled by NF-kB [86], while of fibrinogens, α_2_-macroglobulin, or α_1_-antichymotrypsin strongly depends on STAT3. Regarding individual APP regulation, NF-kB plays an essential role for the transcriptional up-regulation of the SAA [87,88] or AGP expression [89]. Several reports further indicated that the expression of SAA is cooperatively regulated by NF-kB and STAT3 [90,91]. Therefore, understanding the role of APPs in innate immunity, detailed knowledge of its regulations and functions of different proteins, and their mutual interrelationship are required [92].

## Conclusion

Early predictions of economically important diseases including postpartum uterine infections are very important for optimizing productive and reproductive performance in dairy animals. On other hand, estimation of major APPs such as Hp and SAA are seems to be very useful during the transition period for prediction of animals at risk, evaluation of treatment outcome and prognosis of the disease. Although the available results suggested that APPs could differentiate the healthy and inflamed uterus in advance, studies on the cellular and molecular mechanism of APPs regulation during transition or early postpartum period are required. Such studies in naturally infected (with appropriate diagnosis) animals could further strengthen the existing evidence as transition period has differential immune, hormonal, and energy status. Further, it is believed that the Indian cattle are less susceptible or more resistant to diseases due to the strong innate immune system in these animals. Therefore, the correlation between APPs level and postpartum performance during early lactation are needed to be studied in indigenous animals for better understanding.

## Authors’ Contributions

AM, AK, SJ, TKM, VS, and DND conceptualized the concept of this review paper. AM and SJ prepared the final figures and manuscript. NK, SL, MAP, PM, and AA assisted in collecting and compiling the resource material and in manuscript preparation. All authors read and approved the final manuscript.
